# Asthma diagnosis and treatment – 1025. Prevalence of childhood asthma in small city of Iran: an ISAAC study

**DOI:** 10.1186/1939-4551-6-S1-P24

**Published:** 2013-04-23

**Authors:** Shaghayegh Sadat Noorani Hassan Kiadeh, Mohammad Fereidouni, Amir Hossein Khozeime

**Affiliations:** 1 Asthma, Allergy & Immunology Research Center, Birjand University of Medical Sciences, Birjand, Iran

## Background

Asthma and other allergic disorders are common health problems around the world especially in children and have a negative impact on quality of life. Allergic diseases are one of the leading causes of school absence as well as reduction in children performance at school.

Epidemiologic Studies have shown that the prevalence of asthma and other allergic diseases have risen over the past decades. Many factors have been reported that contribute to this increase including genetic factors as well as environmental factors such as lifestyle, infections and diet.

Preventing and controlling allergic diseases require information about the Prevalence, risk factors and triggers which can be vary in different countries. The objective of this study was to determine the prevalence of asthma and other allergic diseases among 6-7 year old children in Birjand city.

## Methods

In a cross-sectional survey in 2011, all school children age 6-7 years in Birjand city were evaluated based on ISAAC protocol. Persian version of ISAAC core questionnaire was completed by parents in total 3070 school children (M/F ratio=0.88) were participated in this study.

## Results

The response rate was 91%. The prevalence rate for Wheezing, physician diagnosis asthma, exercise wheeze, rhinitis and eczema was 15.5%, 2.2%, 3.2%, 12.4% and 8.2% respectively. Except eczema, prevalence of other symptoms was higher in boys than girls but the differences were not significant compared with females.

## Conclusions

Our study shows that prevalence of rhinitis and wheezing is high among school children in Birjand city. Further studies should be performed to determine risk factors of allergic disorders in this area.

